# Is There a Valence-Specific Pattern in Emotional Conflict in Major Depressive Disorder? An Exploratory Psychological Study

**DOI:** 10.1371/journal.pone.0031983

**Published:** 2012-02-20

**Authors:** Zhiguo Hu, Hongyan Liu, Xuchu Weng, Georg Northoff

**Affiliations:** 1 Center for Cognition and Brain Disorders, Hangzhou Normal University, Hangzhou, China; 2 Department of Psychology, Zhejiang Sci-Tech University, Hangzhou, China; 3 Institute of Mental Health Research, University of Ottawa, Ottawa, Canada; Institute of Psychology, Chinese Academy of Sciences, China

## Abstract

**Objective:**

Patients with major depressive disorder (MDD) clinically exhibit a deficit in positive emotional processing and are often distracted by especially negative emotional stimuli. Such emotional-cognitive interference in turn hampers the cognitive abilities of patients in their ongoing task. While the psychological correlates of such emotional conflict have been well identified in healthy subjects, possible alterations of emotional conflict in depressed patients remain to be investigated. We conducted an exploratory psychological study to investigate emotional conflict in MDD. We also distinguished depression-related stimuli from negative stimuli in order to check whether the depression-related distractors will induce enhanced conflict in MDD.

**Methods:**

A typical word-face Stroop paradigm was adopted. In order to account for valence-specificities in MDD, we included positive and general negative as well as depression-related words in the study.

**Results:**

MDD patients demonstrated a specific pattern of emotional conflict clearly distinguishable from the healthy control group. In MDD, the positive distractor words did not significantly interrupt the processing of the negative target faces, while they did in healthy subjects. On the other hand, the depression-related distractor words induced significant emotional conflict to the positive target faces in MDD patients but not in the healthy control group.

**Conclusion:**

Our findings demonstrated for the first time an altered valence-specific pattern in emotional conflict in MDD patients. The study sheds a novel and specific light on the affective mechanisms underlying the abnormal emotional-cognitive interference in MDD. Such emotional conflict bears important clinical relevance since it may trigger the widespread cognitive dysfunctions frequently observed in MDD. The present findings may have important clinical implications in both prediction and psychotherapy of MDD.

## Introduction

Survival adaptation is crucially mediated by emotional response [1]. One central component of emotional response is the monitoring and control of emotional conflict due to interference with ongoing cognitive processing such as working memory and executive functions by task-irrelevant emotionally salient stimuli [2]–[3]. This has been shown to be important for both healthy people and people with clinical mood disorders [2]–[3]. Emotional conflict is often investigated by a word-face Stroop paradigm using faces with negative or positive expressions overlaid with emotional words that are either emotionally incongruent or congruent with the faces (see also [4] for a different paradigm). Subjects are instructed to identify the emotional categorization of the faces [5]–[6] or words [7]–[8] while ignoring the distractor words or faces. Results in these studies consistently show that response to emotionally incongruent (EI) pairs is slower than in emotionally congruent (EC) pairs; this is considered to reflect the effect of emotional conflict. While the psychological correlates of emotional conflict have been well identified in healthy subjects, possible alterations of emotional conflict in depressed patients remain to be investigated.

Patients with major depressive disorder (MDD) suffer from an abnormal imbalance between positive and negative emotions. They remain unable to experience pleasure or positive motivation. Many behavioral studies of depressed individuals have documented significantly diminished responsiveness to positive stimuli (e.g., [9]–[10]). This is in line with the neuroimaging findings showing decreased activation to positive stimuli in the brain in depressed patients relative to control subjects (e.g., [11]), especially in the reward-related areas such as ventral striatum [12] and putamen [13]. These findings are consistent with the apparent anhedonia of depressed patients ([14]–[16]; for a review, see [17]). On the other hand, patients with MDD show enhanced processing of negative stimuli. For example, depressed subjects showed faster responses to negative stimuli than to positive stimuli [18]–[19], and responded slower when the negative material was set as distractor (e.g., [20]–[22]). This is the so-called ‘negative emotional bias’. This means that depressed patients may exhibit a different pattern of emotional conflict when compared to healthy subjects. For example, if positive stimuli are processed abnormally in that they are not perceived as positive in MDD, emotional conflict may remain absent when a negative target is distracted by a positive stimulus. This, however, remains to be tested. Moreover, one may need to distinguish between negative and depression-related items. Some studies observed that depressed patients demonstrated an attentional bias only for depression-related stimuli (e.g., sad faces) from which they could not generalize to other negative stimuli (e.g., angry faces) [19], [23]–[25]. Hence one would expect depression-related stimuli rather than negative ones to induce greater conflict when acting as a distractor. This also remains to be tested.

The aim of the present study was to investigate emotional conflict in MDD. We thereby relied on a well-tested paradigm, the word-face Stroop. In order to account for the above described valence-specificities in MDD, we included positive and general negative as well as depression-related words (e.g., “sad”, “hopeless”) in the study. We predicted a valence-specific pattern in emotional conflict in MDD different from that in healthy subjects.

## Methods

### Participants

This study was approved by the Institute's Ethical Committee of the Center for Cognition and Brain Disorders at Hangzhou Normal University, and all participants gave written informed consent prior to participation.

Twenty out-patients (8 females, 32.0±12.4 years old) from the Mental Health Center of Shantou University, meeting DSM-IV criteria for major depressive disorder, were recruited. Patients were excluded from participation on the basis of the following criteria: history of neurological illness or head injury; alcohol or substance abuse history within the past year; electroconvulsive therapy in the past six months; severe or acute medical conditions. All patients did not take any psychiatric medication within two weeks prior to the current experiment and were thus drug-free at the time of the experiment.

Twenty healthy control subjects (8 females, 32.1±10.1 years old) matched for sex, age, and education level with the MDD patients were recruited by advertisement in the community. They had no history or current diagnosis of any Axis I disorder. All participants (N = 40) were right-handed with normal or corrected-to-normal vision.

Severity of depression in both groups was assessed using the 21-item version of the Beck Depression Inventory (BDI) [26]. Participants of both groups also completed the State-Trait Anxiety Inventory (STAI) [27] consisting of a trait form (STAI-T) and a state form (STAI-S).

### Material

Ninety-six Chinese two-character words were used as distractor stimuli (see the Text S1 for the selection of the words). These consisted of three word types, with 32 words of each type: positive (e.g., “??”(kuai4le4, meaning ‘happy’), “??”(mei3li4, meaning ‘beautiful’)), negative (e.g., “??”(kong3bu4, meaning ‘horrible’), “??”(xiong1e4, meaning ‘vicious’)) and depression-related (e.g., “?”(bei1shang1, meaning ‘sad’), “?”(jue2wang4, meaning ‘hopeless’)). The emotional valence was rated on a 9-point Likert scale (1, most unpleasant, and 9, most pleasant). The mean (±SD) valence of the positive, negative and depression-related words was 7.60(±.49), 2.51(±.45) and 2.43(±.46), respectively. The emotional valence was significantly different between the positive and the other two types (all *p*s<.001). But there was no significant difference between the valence of negative and depression-related words (*p* = .48). Words were selected as depression-relevant if they received a rating of greater than 3 for relevance to depression on a 0∼5 scale (0, not at all related to depression, and 5, highly related to depression). The mean score of relevance to depression of the positive, negative and depression-related words was .28(±.11), 2.02(±.50) and 3.91(±.51), respectively. There were significant differences between each two of the three types (all *p*s<.001). The three word types were matched for frequency and number of strokes of the characters constituting the words (*p*>.05).

Facial expressions were used as target stimuli. Original color face images were selected from the NimStim Face Stimulus Set [28]. Normative ratings of emotional valence and arousal for all facial stimuli were available in a similar procedure described above by 30 additional healthy participants. Two groups of 48 stimuli were created, i.e., positive and negative, based on the rating. The positive group consisted of happy faces from 8 male and 8 female actors, and the negative group involved an equal number of angry, fearful, disgusted and sad faces from the same actors. The emotional valence was significantly different between positive and negative faces, the average valence for positive and negative faces being 7.13(±.39) and 2.74(±.46) (*p*<.001). There was no significant difference between the arousal of the positive and negative faces (6.42(±.64) for positive and 6.25(±.56) for negative faces, *p*>.05).

### Design

**Figure 1:**
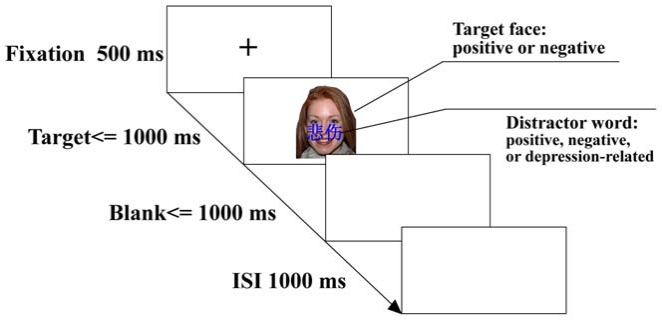
Experimental paradigm. Following a 500 ms warning signal (+), the Stroop stimuli was presented. The distractor word (positive, negative or depression-related) was presented for 200 ms, and the target face (positive or negative) was present for no more than 1000 ms. Subjects were asked to identify the valence of faces while ignoring words overlaid on them within 2000 ms. The target face and the following blank will disappear after the response. The inter-stimulus interval (ISI) was 1000 ms. Emotional incongruent (EI) trials consisted of faces and words with opposing valence, and emotional congruent (EC) trials consisted of faces and words with identical valence. Faces were presented in color and words in blue.

The subjects were instructed to make emotional valence (positive, negative) judgments of faces, ignoring emotional words upon them (see Figure 1). The crossing of distractor word type (positive, negative, depression-related) with target face type (positive, negative) resulted in six experimental conditions, namely, positive distractor word-positive target face (PP), negative distractor-positive target (NP), depression-related distractor-positive target (DP), positive distractor-negative target (PN), negative distractor-negative target (NN), depression-related distractor-negative target (DN). Among them, there were three emotionally congruent conditions, i.e., PP, NN and DN, and three emotionally incongruent conditions, i.e., PN, NP and DP. For each of the six experimental conditions, there were 16 trials. The word and the face in a trial were both randomly selected from the corresponding stimuli set. All types of trials were randomized.

### Procedure

Participants were seated 60 cm in front of a CRT computer monitor. All prompts and stimuli were shown on the CRT using E-prime and participants were asked to respond via keyboard. During the experiment, words and faces were represented at the centre of the CRT, using blue script and colorful facial images against white background. For each trial, a “+” was presented for 500 ms, followed by the combination of a word and a face. The word was presented for 200 ms before it disappeared. The face was presented for no more than 1000 ms. Participants were asked to decide the valence of faces as quickly and accurately as possible upon seeing the face. They could either press “A” or “L” for positive or negative respectively. If a subject responded within 1000 ms, the face would disappear. If not, there would be a blank, which would disappear when a response is made within 1000 ms. The inter-stimulus interval (ISI) is 1000 ms. The two response hands were counterbalanced across subjects.

The participants were instructed to complete the BDI and STAI scales before participating in the emotional categorization task. Participants did a short practice session before the formal experiment. See the Text S2 for supplementary information for the procedure.

### Statistical analysis

As in the previous studies [5]–[8], the effect of emotional conflict was measured by the response difference between the EI condition and EC condition. In our study we used three types of words, positive, general negative and depression-related words as distractors, which not only led to traditional EI (i.e., PN, NP) and EC (i.e., PP, NN) conditions as in the previous studies, but to some special EI (i.e., DP) and EC (i.e., DN) conditions. We defined the emotional conflict effect from the comparison of the traditional EI and EC (i.e., NP-PP, PN-NN) as “*traditional emotional conflict effect*”, and the comparison from the special EI and EC (DP-PP, PN-DN) as “*depression-related emotional conflict effect*”. Accordingly, the statistical analyses were conducted separately in the name of “*traditional emotional conflict effect*” and “*depression-related emotional conflict effect”*. For each of the two types of emotional conflict, a 2 (group: MDD, controls)×2 (target: positive, negative)×2 (congruency: congruent, incongruent) ANOVA on reaction time and error rate data was conducted initially. Then a 2 (target: positive, negative)×2 (congruency: congruent, incongruent) repeated ANOVA was conducted in the MDD and control group separately.

To answer the question whether the effect of emotional conflict was associated with the severity of depression, we examined the relationship between emotional conflict effect and the score of BDI. A regression analysis was performed between the measures of emotional conflict (i.e., PN-NN, PN-DN, NP-PP, DP-PP) and corresponding BDI scores.

## Results

### Group characteristics

Demographic and clinical details for the MDD patients and healthy controls were presented in Table 1. The two groups did not differ significantly with respect to age (*t*(38) = −.03, *p* = .98) and level of education (*t*(38) = −.46, *p* = .65). As expected, there were significant differences between the two groups on the clinical measures: BDI [*t*(38) = 9.24, *p*<.001], STAI-T [*t*(38) = 9.42, *p*<.001] and STAI-S [*t*(38) = 9.97, *p*<.001].

**Table 1:** Demographic and clinical data for MDD patients and healthy controls.

	MDD (N = 20)		Controls (N = 20)	
	M	SD	M	SD
Age (years)	32.0	12.4	32.1	10.1
Education (years)	11.1	4.1	11.7	4.2
BDI	31.5	10.1	6.3	6.8
STAT-T	61.3	10.0	33.5	8.6
STAT-S	60.5	11.6	30.7	6.7

### Reaction time data

The preparation of the data before analysis was described in the Text S3. Consistent with the statistical analysis, the results were reported under the title of “*traditional emotional conflict effect*” and “*depression-related emotional conflict effect*” separately.

#### Traditional emotional conflict effect

Mean response times and error rates were initially analyzed by way of repeat-measure ANOVAs with group (MDD, controls) as between-subject factor, and target valence (positive, negative) and emotional congruency (congruent, incongruent) as within-subject factors. The mean reaction time (RT) and error rate for each condition were shown in Table 2.

**Table 2:** Mean reaction times (ms) and error rates (% in brackets) for traditional emotional conflict effect in MDD patients and healthy controls.

	Negative Target			Positive Target		
	NN	PN	PN-NN	PP	NP	NP-PP
MDD	749 (5.6)	773 (15.3)	25	738 (7.2)	770 (9.4)	32
Controls	634 (8.4)	687 (11.6)	52**	611(4.7)	623 (5.9)	12

*Note.*

**p<.01. Abbreviations: NN represents a combination of negative word distractor and negative face target; PN, positive distractor – negative target; PP, positive distractor – positive target; NP, negative distractor – positive target.

Results on RT revealed a significant main effect of group [*F*(1,38) = 12.79, *p*<.001], target valence [*F*(1,38) = 6.89, *p*<.05] and congruency [*F*(1,38) = 11.21, *p*<.01]. The group×target valence×congruency interaction also approached significance [*F*(1,38) = 4.72, *p*<.05], but no interaction between any two factors approached significance (all *p*s>.05).

**Figure 2:**
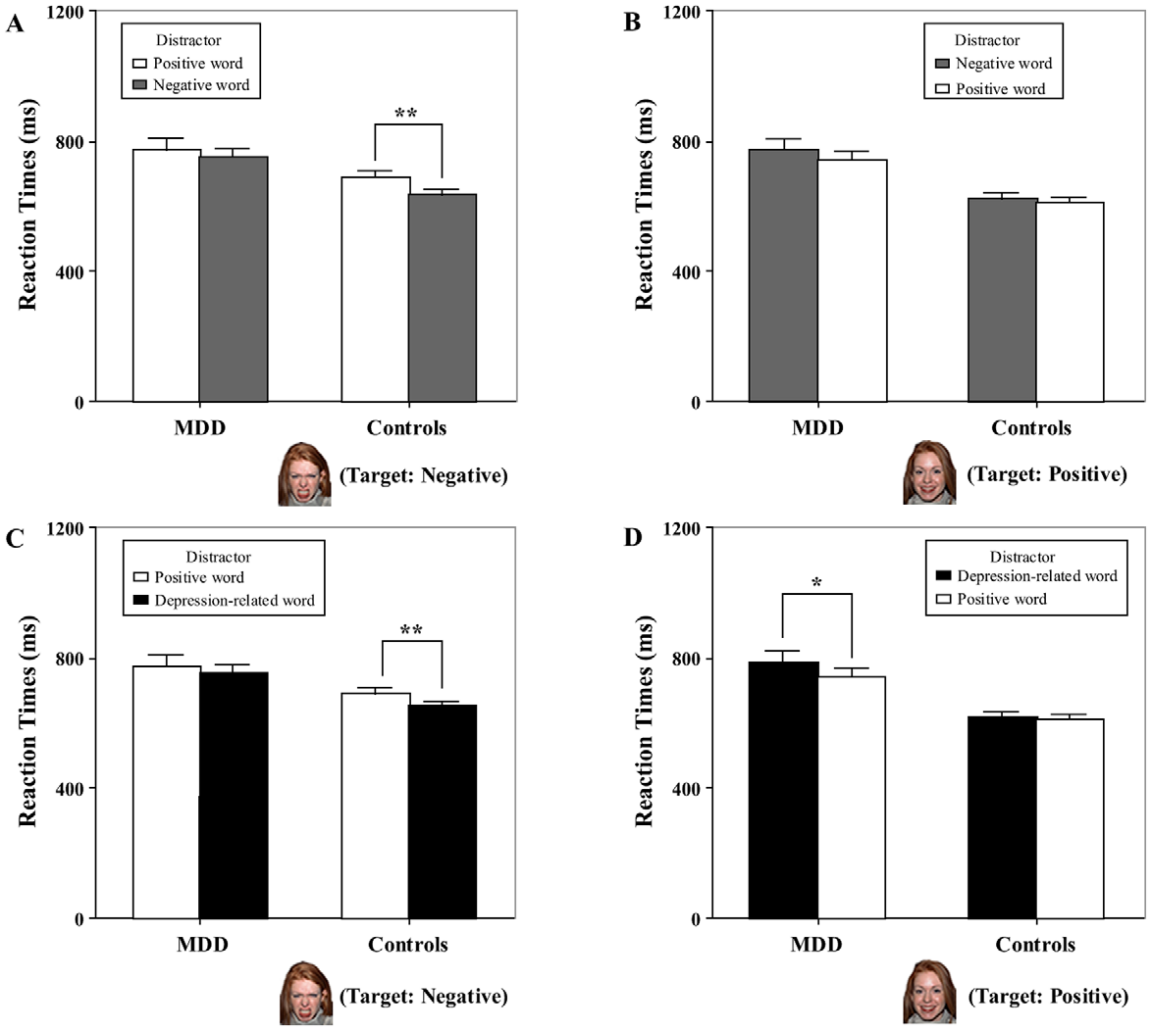
Comparisons of effects of emotional conflict between MDD patients and healthy controls. The emotional conflict effect induced by the traditional emotional incongruent (EI) trials (i.e., positive word distractor and negative face target, negative distractor - positive target) and emotional congruent (EC) trials (i.e., positive distractor - positive target, negative distractor - negative target) was illustrated in (A) (negative target) and (B) (positive target). The emotional conflict effect induced by the special EI (i.e., depression-related distractor - positive target) trials and EC (i.e., depression-related distractor - negative target) trials associated with the depression-related words was shown in (C) (negative target) and (D) (positive target). Error bars show standard errors. * p<.05, ** *p*<.01.

A 2 (target valence: positive, negative)×2 (congruency: congruent, incongruent) repeated ANOVA on RTs was conducted in the MDD and healthy group, separately. In the MDD group, analyses showed no significant main effect of target valence [*F*(1,19) = .19, *p* = .67] and congruency [*F*(1,19) = 3.23, *p* = .09], neither did the valence by congruency interaction approach significance [*F*(1,19) = .17, *p* = .69] (see left part of Figure 2A and 2B). In the healthy control group, by contrast, analyses demonstrated a significant main effect of target valence [*F*(1,19) = 17.11, *p*<.001] and congruency [*F*(1,19) = 13.18, *p*<.01], and a significant interaction effect between the valence and congruency [*F*(1,19) = 9.58, *p*<.01]. Post hoc analyses indicated that the congruency effect was significant when the target was negative [*F*(1,19) = 14.40, *p*<.001] (see right part of Figure 2A), while the congruency effect did not approach significance when the target was positive [*F*(1,19) = 2.74, *p* = .11] (see right part of Figure 2B).

#### Depression-related emotional conflict effect

Similar analyses with aforementioned were conducted, except that the negative word distractor was replaced by the depression-related word distractor, i.e., the NP was replaced by DP, and NN was replaced by DN. For mean reaction time and error rate for each condition see Table 3.

**Table 3:** Mean reaction times (ms) and error rates (% in brackets) for depression-related emotional conflict effect in MDD patients and healthy controls.

	Negative Target			Positive Target		
	DN	PN	PN-DN	PP	DP	DP-PP
MDD	749 (7.5)	773 (15.3)	24	738 (7.2)	783 (13.8)	45*
Controls	649 (5.6)	687 (11.6)	38**	611 (4.7)	617 (6.9)	6

*Note.*

*p<.05,

**p<.01. Abbreviations: DN represents a combination of depression-related word distractor and negative face target; PN, positive distractor – negative target; PP, positive distractor – positive target; DP, depression-related distractor – positive target.

Results on RT revealed a significant main effect of group [*F*(1,38) = 12.19, *p*<.001], target valence [*F*(1,38) = 7.27, *p*<.01] and congruency [*F*(1,38) = 9.84, *p*<.01]. The group×target valence×congruency interaction also approached significance [*F*(1,38) = 6.28, *p*<.05]. The group×target valence interaction also approached significance [*F*(1,38) = 6.90, *p*<.05], but no other interaction approached significance (all *p*s>.05).

A 2 (target valence: positive, negative)×2 (congruency: congruent, incongruent) repeated ANOVA on RTs was conducted in the MDD and healthy group separately. In the MDD group, analyses showed a significant main effect of congruency [*F*(1,19) = 4.39, *p*<.05], but no significant main effect of target valence [*F*(1,19) = .002, *p* = .97], neither did the valence by congruency interaction approach significance [*F*(1,19) = 1.48, *p* = .24]. Follow-up analyses indicated that the congruency effect was not significant when the target was negative [*F*(1,19) = 2.26, *p* = .15] (see left part of Figure 2C), while the congruency effect approached significance when the target was positive [*F*(1,19) = 4.63, *p*<.05] (see left part of Figure 2D), suggesting a longer reaction time in DP condition relative to PP in MDD. In the healthy group analyses showed a significant main effect of target valence [*F*(1,19) = 18.89, *p*<.001] and congruency [*F*(1,19) = 9.42, *p*<.01] and a significant interaction effect between the valence and congruency [*F*(1,19) = 7.02, *p*<.05]. Post hoc analyses indicated that the congruency effect was significant when the target was negative [*F*(1,19) = 18.33, *p*<.001] (see right part of Figure 2C), while the congruency effect did not approach significance when the target was positive [*F*(1,19) = .37, *p* = .55] (see right part of Figure 2D).

Analysis of error rate showed no speed–accuracy trade-off (See Text S4 for details).

### Regression analysis

**Figure 3:**
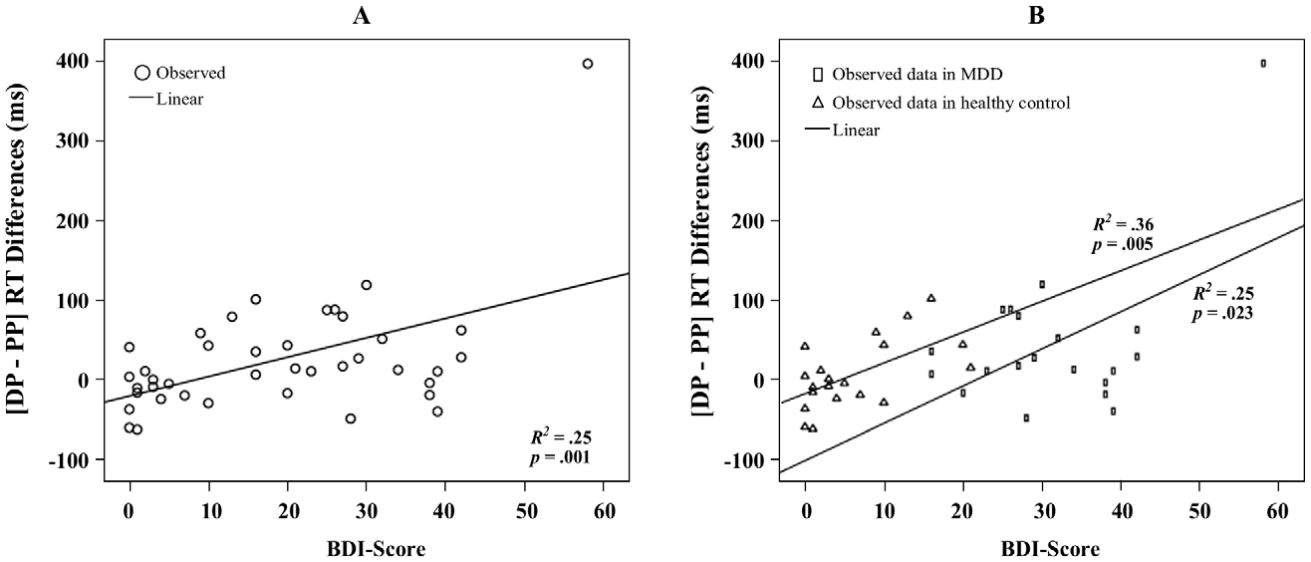
Illustration of the regression analysis between the depression-related emotional conflict effect of [DP-PP] and BDI score. (A) Data across MDD and healthy control groups. (B) Data in MDD group and healthy group separately. Abbreviations: DP represents a combination of depression-related word distractor and positive face target; PP, positive distractor - positive target.

Regression analysis showed the relationship between emotional conflict effect and the score of BDI. Results demonstrated that the global linear fit of the analysis was significant in [DP-PP] across all the subjects [*R* ^2^ = .25, *F*(1,38) = 12.56, *p*<.001] (see Figure 3A) and also significant in both MDD group [*R* ^2^ = .25, *F*(1,18) = 6.13, *p*<.05] and healthy group [*R* ^2^ = .36, *F*(1,18) = 10.08, *p*<.01] (see Figure 3B). The global linear fit did not approach significance in either [PN-NN][*R* ^2^ = .00, *F*(1,38) = .01, *p* = .93] or [PN-DN] [*R* ^2^ = .00, *F*(1,38) = .00, *p* = .98]. The global linear fit of the analysis was marginally significant in [NP-PP] [*R* ^2^ = .09, *F*(1,38) = 4.00, *p* = .054] (see the Figure S1). Other details of the regression results can be found in the Text S5.

## Discussion

This study investigated emotional conflict in out-patients diagnosed with MDD. We also distinguished depression-related stimuli from negative stimuli in order to check whether the depression-related distractors will induce enhanced conflict in MDD. The main findings of our study are: (1) There are significant changes in the pattern of emotional conflict in MDD patients when compared to the healthy control group. (2) The positive distractor words could not induce significant emotional conflict to the negative target faces in MDD patients while they could in healthy subjects. (3) The depression-related distractor words induced significant emotional conflict to the positive target faces in MDD patients while no such conflict was found in the healthy group. In this way, MDD patients were clearly distinguishable from the healthy group.

Our main overall finding consists in significant differences in the valence-specific pattern of emotional conflict in MDD. This concerned especially the positive items. While positive distractors (contrary to negative distractors) induced significant conflict effect to negative targets in healthy subjects, this effect did not reach significance in MDD patients. Moreover, these findings in “*traditional emotional conflict effect*” (see Figure 2A) were replicated in “*depression-related emotional conflict effect*” (see Figure 2C). In contrast to healthy subjects, MDD subjects did not demonstrate any conflict effect or longer reaction times during the presentation of negative faces overlaid by positive words (contrary to negative or depression-related words). Taken together, these findings argue in favor of a specific deficit in the processing of positive emotions in MDD patients.

More specifically, one would assume that the positive emotions are no longer processed as positive emotions and henceforth no longer induce emotional conflict in MDD. This interpretation could also explain our finding that responses to positive faces were not significantly interfered by negative distractors in MDD (see Figure 2B). Our results are in line with other findings also showing MDD to be a disorder of specifically positive emotional processing [9]–[10] and evidences of dysfunctions of the reward system in MDD [12]–[13]. This is well reflected in what has long been described as anhedonia—the inability to experience pleasure in depressed patients ([14]–[16]; see [17] for a review). Since the BDI score is strongly driven by items on anhedonia [16], our results lend further evidence to the central role of a deficit in positive emotional processing underlying altered emotional conflict in MDD.

Opposite to depressed patients, healthy subjects showed a bias toward positive information ([29]–[31], see [25] for a review). This could also explain the non-significant effect of emotional conflict when the target consisted of positive faces in healthy subjects (see Figure 2B and 2D).

In addition to the positive emotional valence alterations, MDD patients showed a specific emotional conflict associated with depression-related items. That is, the depression-related words, contrary to the positive ones, induced significantly greater interference effects to positive target faces in MDD patients whereas this effect did not occur in healthy subjects (see Figure 2D). This is especially remarkable given the fact that such an effect could not be obtained for negative words (see Figure 2B). This lets one suggests that the depressed items must be distinguished from the negative items. This is consistent with the findings of attentional bias in MDD towards depression related information (e.g., sad) [19], [23]–[25], [32] but not the other negative information (e.g., angry) [30], [33]–[35]. This result was further supported by the regression analysis showing that subjects (depressed and healthy) with higher BDI scores exhibited increased effect of emotional conflict induced by depression-relevant distractors (see Figure 3). Our findings suggested that the emotional conflict induced by depression-related items could be used as a predictor of severity of depressive symptoms.

More generally, the present results mean that MDD cannot be characterized as a disorder of negative emotional processing. Instead, as discussed above, one may regard MDD rather a disorder of positive emotional processing. However, further investigations are necessary to better understand the relationship between the apparent deficit in positive emotional processing and the depression-related items used here (see the Supporting Information Text S6 for the correlation analysis between the positive-negative interference and the depression-related-positive interference in both MDD and control group). This in turn may provide a better understanding of the underlying deficit driving depression and its clinical symptoms like anhedonia and depression-related bias that may constitute the clinical manifestation of what we tested as emotional conflict.

Our findings of the strong impact of the distractor suggest an implicit or unconscious processing change in MDD. More specifically, the balance between positive and negative emotional processing may already be altered in MDD in the pre-conscious stage of emotion processing [36]. This would indicate, however, that psychotherapeutic strategies targeting conscious emotion regulation may miss such pre- or un-conscious emotion processing deficit. Future psychotherapeutic strategies that target the personal relevance encoded in such un- or pre-conscious emotional processing may be needed. This has in part been addressed by psychodynamic therapeutic strategies [36]. These though remain currently nonspecific to, for instance, re-balance the apparently abnormal negative-positive dysbalance and the exceptionally high sensitivity to distractors, resulting in the here observed emotional conflict.

Emotion-cognition regulation has often been investigated in both healthy subjects and MDD patients, showing involvement and alteration of mainly lateral (and in part medial) prefrontal cortical regions and the amygdala, with the former top-down modulating the latter (see the works by Ochsner and Gross as for instance [37]). The observed deficits in emotional conflict seem to argue rather in favor of abnormal bottom-up modulation from, for instance, subcortical regions like the raphe nucleus to anterior cortical midline regions like the sub- and pre-genual anterior cingulated cortex [38]–[40]. While such bottom-up modulation has been well demonstrated in animal models of MDD (see [41]), future studies in humans are needed to associate such subcortical-cortical midline bottom-up modulation with implicit, i.e., pre- or un-conscious emotional processing of the negative-positive valence balance.

One limitation of the present study is the relatively limited number of subjects (20 MDD and 20 controls). It is important to bear in mind that the small size of the sample could sometimes lead to the non-significance of the differences, which does not necessarily mean that the effect of emotional conflict does not exist. The low number of subjects in especially MDD, combined with the novel design concerning the first-time inclusion of such type depression-related stimuli and emotional conflict, led us to characterize our study as exploratory. Future studies are needed to further validate this paradigm also in MDD patients with a larger number of subjects.

In conclusion, we report here about an altered valence-specific pattern in emotional conflict in MDD patients. This concerns especially the positive emotional information and depression-related stimuli as distinguished from negative emotional stimuli. Our exploratory study thus sheds a novel and more specific light on the affective mechanisms underlying the abnormal emotional-cognitive interference in MDD. It potentially bears important clinical relevance in both prediction and psychotherapy of MDD.

## Supporting Information

Figure S1 Illustration of the regression analysis between the traditional emotional conflict effect of [NP-PP] and BDI score. (A) Data across MDD and healthy control groups. (B) Data in MDD group and healthy group separately. Abbreviations: NP represents a combination of negative word distractor and positive face target; PP, positive distractor - positive target.
(TIF)

Text S1 The selection of the distractor words.
(DOC)

Text S2 Supplementary procedure.
(DOC)

Text S3 Preparation of the data.
(DOC)

Text S4 Results of error rate data.
(DOC)

Text S5 Supplementary results of regression analysis.
(DOC)

Text S6 Correlation analysis between the positive-negative interference and the depression-related-positive interference.
(DOC)
