# Supplementary figures and images for: Is There a Valence-Specific Pattern in Emotional Conflict in Major Depressive Disorder? An Exploratory Psychological Study
Source: PLoS One. 2012 Feb 20;7(2):e31983. doi: 10.1371/journal.pone.0031983 (PMC3282781; doi:10.1371/journal.pone.0031983)

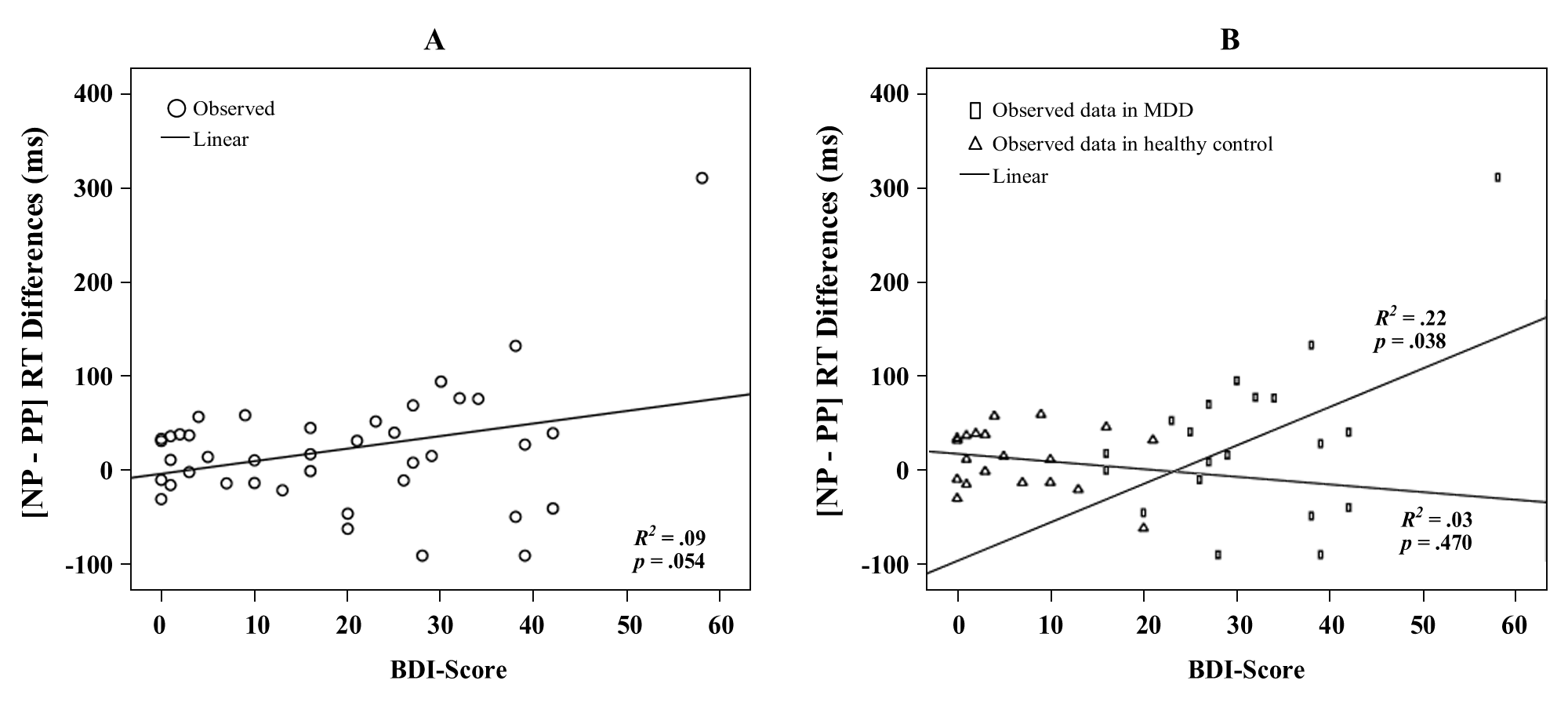

Supplement: Figure S1 — Illustration of the regression analysis between the traditional emotional conflict effect of [NP-PP] and BDI score. (A) Data across MDD and healthy control groups. (B) Data in MDD group and healthy group separately. Abbreviations: NP represents a combination of negative word distractor and positive face target; PP, positive distractor - positive target. (TIF) [file pone.0031983.s001.tif]
