# Supplementary material for: Is There a Valence-Specific Pattern in Emotional Conflict in Major Depressive Disorder? An Exploratory Psychological Study
Source: PLoS One. 2012 Feb 20;7(2):e31983. doi: 10.1371/journal.pone.0031983 (PMC3282781; doi:10.1371/journal.pone.0031983)
Supplement: Text S1 — The selection of the distractor words. (DOC) [file pone.0031983.s002.doc]

**The selection of the distractor words**

The selection of the distractor words was based on ratings of the emotional valence and the relevance to depression by 20 additional healthy participants (who were not participants of the formal experiment). The subjects were told the symptoms and features of depression and what the depression-related words are like before the rating.
