# Supplementary material for: Is There a Valence-Specific Pattern in Emotional Conflict in Major Depressive Disorder? An Exploratory Psychological Study
Source: PLoS One. 2012 Feb 20;7(2):e31983. doi: 10.1371/journal.pone.0031983 (PMC3282781; doi:10.1371/journal.pone.0031983)
Supplement: Text S2 — Supplementary procedure. (DOC) [file pone.0031983.s003.doc]

**Supplementary procedure**

The experiment was conducted under identical light and sound controlled conditions. The apparatus for the experiment consisted of a keyboard and a CRT, a Windows PC that controlled stimulus presentation and response recording using E-prime (Psychology Software Tools, Inc., Pittsburgh, PA) with millisecond timing accuracy.

The distractor words were shown in blue with Song font (bold, size = 48 points). The color facial images were cropped to the same size, subtending approximately 8º of visual angle vertically and 6° horizontally.
