# Supplementary material for: Is There a Valence-Specific Pattern in Emotional Conflict in Major Depressive Disorder? An Exploratory Psychological Study
Source: PLoS One. 2012 Feb 20;7(2):e31983. doi: 10.1371/journal.pone.0031983 (PMC3282781; doi:10.1371/journal.pone.0031983)
Supplement: Text S3 — Preparation of the data. (DOC) [file pone.0031983.s004.doc]

**Preparation of the data**

Before analysis of the data all trials with incorrect response were omitted. Next, responses beyond three standard deviations from the mean were considered as outliers, reflecting anticipatory or delayed responding, and were also removed from analyses. The number of outliers accounted for 0.6% of the total number of trials in the depressed group, and 0.4% in the control group, and they did not differ between the two groups (*p* = .49).
