# Supplementary material for: Is There a Valence-Specific Pattern in Emotional Conflict in Major Depressive Disorder? An Exploratory Psychological Study
Source: PLoS One. 2012 Feb 20;7(2):e31983. doi: 10.1371/journal.pone.0031983 (PMC3282781; doi:10.1371/journal.pone.0031983)
Supplement: Text S4 — Results of error rate data. (DOC) [file pone.0031983.s005.doc]

**Results of error rate data**

*Traditional emotional conflict effect*

A group (MDD, controls) × target valence (positive, negative) × congruency (congruent, incongruent) ANOVA on errors revealed a significant main effect of target valence [*F*(1,38) = 5.15, *p* < .05] and congruency [*F*(1,38) = 7.68, *p* < .01], but no significant main effect of group [*F*(1,38) = .69, *p* = .41]. The group × target valence × congruency interaction did not approach significance [*F*(1,38) = .76, *p* = .39].

*Depression-related emotional conflict effect*

A similar group × target valence × congruency ANOVA on error rates revealed a significant main effect of congruency [*F*(1,38) = 14.42, *p* < .001], but no significant main effect of group [*F*(1,38) = 2.12，*p* = .15] and target valence [*F*(1,38) = 1.01, *p* = .32]. The group × target valence × congruency interaction did not approach significance [*F*(1,38) = .23, *p* = .63].
