# Supplementary material for: Is There a Valence-Specific Pattern in Emotional Conflict in Major Depressive Disorder? An Exploratory Psychological Study
Source: PLoS One. 2012 Feb 20;7(2):e31983. doi: 10.1371/journal.pone.0031983 (PMC3282781; doi:10.1371/journal.pone.0031983)
Supplement: Text S5 — Supplementary results of regression analysis. (DOC) [file pone.0031983.s006.doc]

**Supplementary results of regression analysis**

Regression analysis showed the relationship between emotional conflict effect and the score of BDI. In the MDD group, results showed that the global linear fit of the analysis was significant in [NP-PP] [*R*^2^ = .22, *F*(1,18) = 5.03, *p* < .05] (see Figure S1B), while the global linear fit did not approach significance in [PN-NN][ *R*^2^ = .13, *F*(1,18) = 2.76, *p* = .11] or [PN-DN] [*R*^2^ = .05, *F*(1,18) = 1.00, *p* = .33]. In the healthy group, results showed that the global linear fit of the analysis was not significant in [NP-PP] [*R*^2^ = .03, *F*(1,18) = .55, *p* = .47] (see Figure S1B), [PN-NN] [*R*^2^ = .03, *F*(1,18) = .60, *p* = .45] or [PN-DN] [*R*^2^ = .01, *F*(1,18) = .11, *p* = .74].
