# Supplementary material for: Is There a Valence-Specific Pattern in Emotional Conflict in Major Depressive Disorder? An Exploratory Psychological Study
Source: PLoS One. 2012 Feb 20;7(2):e31983. doi: 10.1371/journal.pone.0031983 (PMC3282781; doi:10.1371/journal.pone.0031983)
Supplement: Text S6 — Correlation analysis between the positive-negative interference and the depression-related-positive interference. (DOC) [file pone.0031983.s007.doc]

**Correlation analysis between the positive-negative interference and the depression-related-positive interference**

We calculated the correlation between the positive-negative interference and the depression-related-positive interference in both MDD and control group. The correlation between the RT differences of [PN-NN] and [DP-PP] is significant (*r* = .68, *p* < .001), as well as the correlation between [PN-DN] and [DP-PP] (*r* = .59, *p* < .01) in MDD. However, in the control group the correlation between the RT differences of [PN-NN] and [DP-PP] is not significant (*r* = 0.43, *p =* .055), neither is the correlation between [PN-DN] and [DP-PP] (*r* = 0.17, *p* = .480). These results indicated that the positive processing deficit in MDD may contribute to the depression-related emotional conflict found in the present study.
